# A new protocol for the preparation of superconducting KBi_2_

**DOI:** 10.1039/d0ra04541a

**Published:** 2020-07-16

**Authors:** Huan Li, Yanan Wang, Yutaro Aoki, Saki Nishiyama, Xiaofan Yang, Tomoya Taguchi, Akari Miura, Ai Suzuki, Lei Zhi, Hidenori Goto, Ritsuko Eguchi, Takashi Kambe, Yen-Fa Liao, Hirofumi Ishii, Yoshihiro Kubozono

**Affiliations:** Research Institute for Interdisciplinary Science, Okayama University Okayama 700-8530 Japan kubozono@cc.okayama-u.ac.jp; Department of Physics, Okayama University Okayama 700-8530 Japan; National Synchrotron Radiation Research Center Hsinchu 30076 Taiwan

## Abstract

A superconducting KBi_2_ sample was successfully prepared using a liquid ammonia (NH_3_) technique. The temperature dependence of the magnetic susceptibility (*M*/*H*) showed a superconducting transition temperature (*T*_c_) as high as 3.6 K. In addition, the shielding fraction at 2.0 K was evaluated to be 87%, *i.e.*, a bulk superconductor was realized using the above method. The *T*_c_ value was the same as that reported for the KBi_2_ sample prepared using a high-temperature annealing method. An X-ray diffraction pattern measured based on the synchrotron X-ray radiation was analyzed using the Rietveld method, with a lattice constant, *a*, of 9.5010(1) Å under the space group of *Fd*3̄*m* (face-centered cubic, no. 227). The lattice constant and space group found for the KBi_2_ sample using a liquid NH_3_ technique were the same as those reported for KBi_2_ through a high-temperature annealing method. Thus, the superconducting behavior and crystal structure of the KBi_2_ sample obtained in this study are almost the same as those for the KBi_2_ sample reported previously. Strictly speaking, the magnetic behavior of the superconductivity was different from that of a KBi_2_ sample reported previously, *i.e.*, the KBi_2_ sample prepared using a liquid NH_3_ technique was a type-II like superconductor, contrary to that prepared using a high-temperature annealing method, the reason for which is fully discussed. These results indicate that the liquid NH_3_ technique is effective and simple for the preparation of a superconducting KBi_2_. In addition, the topological nature of the superconductivity for KBi_2_ was not confirmed.

## Abstract 2

A superconducting KBi_2_ sample was successfully prepared using a liquid ammonia (NH_3_) technique.

## Graphical abstract

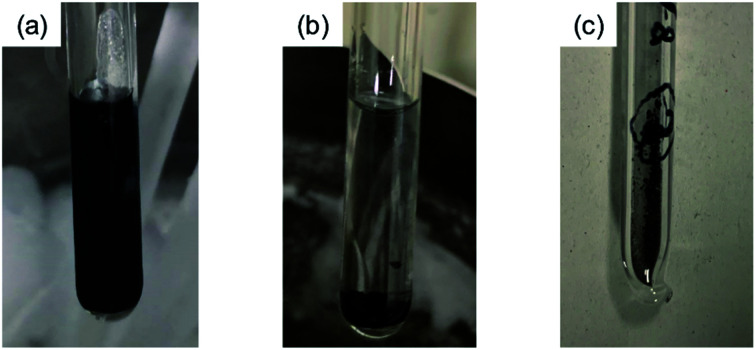

## 1. Introduction

Topological materials such as a topological insulators, Dirac and Weyl semimetals, and axion insulators have attracted significant attention from physicists and chemists owing to their exciting and fascinating physical properties.^1–29^ Gapless surface states (Dirac-like linear dispersions) in a topological insulator are protected through a time reversal symmetry, which show a spin polarization to lock the spin along a direction perpendicular to the momentum (spin-momentum locking).^1–5^ Here, it is noted that the bulk of a topological insulator possesses a band gap, as in a traditional insulator. The representative topological insulators are Bi_1−*x*_Sb_*x*_, Bi_2_Se_3_, Bi_2_Te_3_, and Sb_2_Te_3_,^6–10^ with Bi_2_Se_3_ being the most popular three-dimensional (3D) topological insulator, having a single Dirac cone inside a band gap at the center of the Brillouin Zone (BZ).^7,8^

Moreover, Dirac and Weyl semimetals have also elicited attention from physicists and chemists owing to their unique electronic structures, in which linear band crossings are in fourfold/twofold degenerated points.^11–15^ A Dirac semimetal appears when both time-reversal symmetry and special inversion symmetry are maintained, whereas a Weyl semimetal appears when either the time-reversal symmetry or special inversion symmetry is broken. Namely, by breaking either the time-reversal symmetry or the special inversion symmetry, one Dirac fermion will transform into two Weyl fermions with opposite chirality in the BZ. The above semimetals are categorized as type-I or type-II depending on whether the Lorentz invariance is preserved. With type-I Dirac/Weyl semimetals, massless Dirac fermions (or Weyl fermions) should emerge at the Dirac and Weyl points,^15–19^ whereas in type-II Dirac/Weyl semimetals, they emerge at the topologically protected crossing points of the electron and hole pockets.^17,20–22^ Currently, many researchers are working on the condensed matter physics of Dirac/Weyl semimetals, owing to not only the above unique electronic properties, but also interesting physical properties such as a negative magnetoresistance,^23,24^ chiral magnetic effects,^25^ and quantum anomalous Hall (QAH) effect.^26^ Furthermore, recent interest in the complex interplay between topology and magnetism has led to a topological quantum state, which is indicated as an axion insulator.^27,28^ The attempt to realize an axion insulator began in the heterostructures of QAH effect films,^27^ and is currently realized in a stoichiometric material.^28^

Alkali–Bi based compounds (ABi_3_ (A = Na, K, or Rb)) have recently attracted significant attention because crystalline ABi_3_ has been proposed as a Dirac semimetal in which bulk 3D Dirac points are protected by a crystal symmetry.^15,29^ Moreover, these materials were predicted to possess a nontrivial Fermi arc on the surface. In addition, various novel physical properties (giant diamagnetism, linear quantum magnetoresistance, and the QAH effect) were expected for ABi_3_.^29^ Since the above reports, the physical properties of a family of materials (alkaline and alkaline-earth metal–Bi compounds) have been investigated owing to an interest in the coupling of a Dirac semimetal and the superconductivity, *i.e.*, under the topological nature of the superconductivity, the particular superconducting properties of BaBi_3_ were fully investigated.^30^ In 2016, the superconductivity of the KBi_2_ sample, which was a traditional superconductor,^31^ was fully studied.^32^ The detailed data of the superconductivity in KBi_2_ were collected in this study. The motivation of this study is the interplay of the topological nature and the superconductivity. In addition, the strong spin orbit coupling (SOC) may affect the superconductivity in a metal–Bi compound because Bi is a heavy atom (atomic no. 83). All metal–Bi compounds were prepared using a high-temperature annealing method,^30–35^ for instance, a stoichiometric amount of Bi and K were mixed and sealed in a quartz tube. The tube was heated at more than 500 °C for several hours and slowly cooled. Finally, the crystals of KBi_2_ were isolated using a centrifuge. Thus, the heating of metal and Bi is indispensable for the preparation of metal–Bi compounds, and isolation using a centrifuge is frequently required.

In this study, we report a new protocol for the preparation of KBi_2_ in which a sample heating is not employed, *i.e.*, the liquid ammonia (NH_3_) technique was applied to the sample preparation of a superconducting KBi_2_. The KBi_2_ sample obtained using liquid NH_3_ showed a bulk superconductivity, and a clear crystalline powder was prepared. To characterize its superconductivity and crystal structure, the magnetic susceptibility (*M*/*H*) and X-ray diffraction (XRD) pattern of the obtained sample were measured; *M* and *H* refer to the magnetization and applied magnetic field, respectively. The superconducting transition temperature, *T*_c_, and crystal structure of the KBi_2_ sample prepared using liquid NH_3_ were consistent with those of the sample prepared through a high-temperature annealing method. However, the magnetic behavior of the superconductivity was significantly different from that of the KBi_2_ sample reported previously,^32^ which is fully discussed herein.

## 2. Experimental

Stoichiometric amounts of K and Bi were inserted into a glass tube in an Ar-filled glove box, and the space inside the glass tube was pumped to 10^−2^ torr. NH_3_ gas was then collected in a glass tube and frozen. The K was dissolved in liquid NH_3_ at less than −50 °C, and K atoms were introduced into the Bi crystallites, forming KBi_2_. Finally, NH_3_ was removed through vacuum distillation, and the removal of NH_3_ was then carried out through pumping under 10^−2^ torr at room temperature. Because the KBi_2_ sample is quite sensitive to air, the handling of the sample was achieved without air exposure. Details of the reaction process are described later.

The temperature dependence of *M*/*H* of the obtained KBi_2_ sample was measured using a SQUID magnetometer (Quantum Design, MPMS2 or MPMS–SQUID–VSM). The powder XRD pattern of the sample was measured at room temperature, using synchrotron radiation at BL12B2 of SPring-8, and the wavelength *λ* of the X-ray beam was 0.6859610 Å. The XRD pattern was analyzed using a Rietveld refinement.

## 3. Results and discussion

**Fig. 1:**
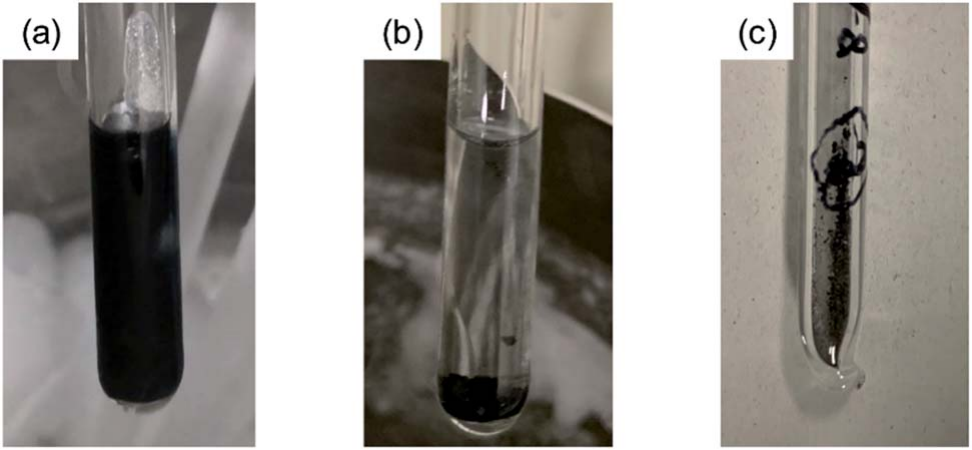
Process of KBi_2_ sample formation. Photographs of (a) the first stage where K metal dissolves in liquid NH_3_, and (b) the final stage where KBi_2_ is formed. In the first stage, the color of the liquid NH_3_ solution is dark blue, and in the final stage, the color of the solution is transparent. (c) Photograph of KBi_2_ sample obtained after the removal of NH_3_.

Fig. 1 shows the process of the sample preparation of KBi_2_. A photograph of the initial stage of the reaction between K and Bi is shown in Fig. 1(a), and K is dissolved in liquid NH_3_. The color of the K-dissolved NH_3_ solution is dark blue. After 3 days, the solution became transparent and black precipitate was obtained, as shown in Fig. 1(b). After removal of NH_3_, a black powder sample was obtained, as shown in Fig. 1(c). The sample was then introduced into a quartz cell and glass capillary for *M*/*H* – *T* and XRD measurements, respectively, in an Ar-filled glove box. Here, it should be stressed that the most important point in the sample preparation of KBi_2_ is to handle the sample without exposure to air to avoid a degradation of the sample.

**Fig. 2:**
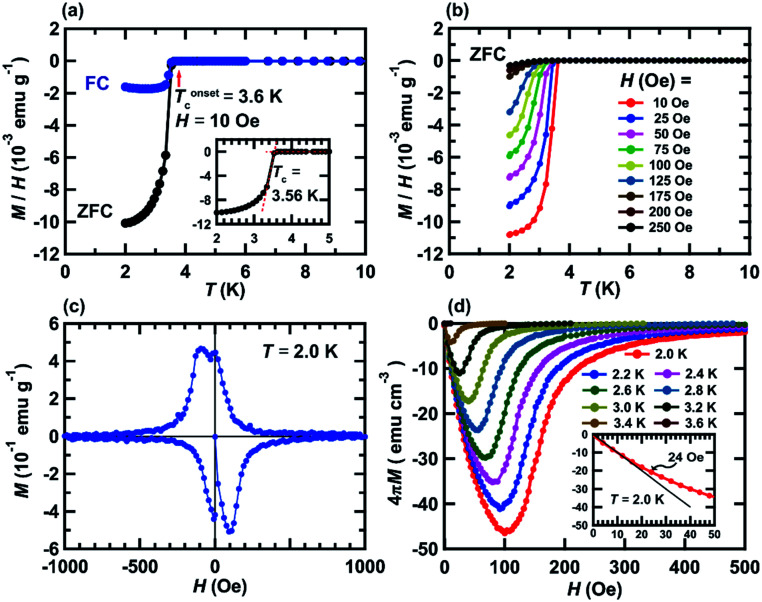
(a) *M*/*H* – *T* plots recorded in ZFC and FC modes (*H* = 10 Oe), (b) *M*/*H* – *T* plots in ZFC modes at *H* = 10–250 Oe, (c) *M* – *H* loop recorded at 2.0 K and (d) 4π*M* – *H* plots at 2.0–3.6 K. The measured sample is KBi_2_ prepared using a liquid NH_3_ technique. The definition of *T*_c_ is shown in the inset of (a). The method for determining *H*_c1_ is shown in the inset of (d).

Fig. 2(a) shows *M*/*H* – *T* plots of the KBi_2_ sample obtained using the liquid NH_3_ technique in zero-field cooling (ZFC) and field-cooling (FC) measurement modes; the value of *H* was 10 Oe. As shown in the inset of Fig. 2(a), the superconducting transition temperature, *T*_c_, was determined to be 3.56 K from the crossing point between the dropped *M*/*H* – *T* plot and that under a normal state. In addition, the onset superconducting transition temperature, *T*^onset^_c_, was 3.6 K (Fig. 2(a)). The values of *T*_c_ and *T*^onset^_c_ determined from the *M*/*H* – *T* plot in ZFC mode were the same as those from the *M*/*H* – *T* plot in FC mode. The values of *T*_c_ and *T*^onset^_c_ were the same as those of the KBi_2_ sample prepared using a high-temperature heating method.^32^ The shielding fraction at 2.0 K determined from the *M*/*H* – *T* plot in ZFC mode was evaluated to be 87%, indicating a bulk superconductivity.

**Fig. 3:**
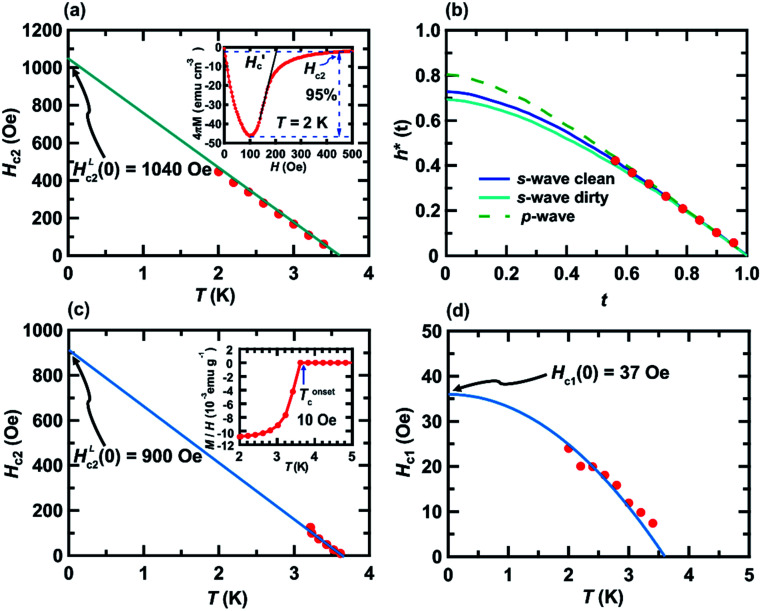
(a) *H*_c2_ – *T*, (b) h* – *t*, (c) *H*_c2_ – *T*, and (d) *H*_c1_ – *T* plots of KBi_2_ sample prepared using liquid NH_3_ technique. Definition of all parameters in (a)–(d) are fully shown in the text. The method for determining the *H*_c2_ value at each *T* is shown in the inset of (a). Inset of (c), the *M*/*H* – *T* plot at 10 Oe shown in Fig. 2(b) is provided to show the *T*^onset^_c_ value, as an example. The value of *T*^onset^_c_ was utilized to depict the *H*_c2_ – *T* plot in (c).

The *M*/*H* – *T* plots at *H* = 10–250 Oe in ZFC mode are shown in Fig. 2(b), exhibiting a clear suppression of the superconducting transition by applying *H*. The *T*^onset^_c_ value against each *H* is employed for depicting the *H* – *T* phase diagram (Fig. 3(c)), as described later. Fig. 2(c) shows the *M* – *H* loop of a KBi_2_ sample prepared using a liquid NH_3_ technique, recorded at 2.0 K, showing a clear hysteresis loop. This behavior indicates a type-II superconductor, which is different from that (type-I superconductor) reported previously for a KBi_2_ crystal,^32^ which was prepared using a high-temperature annealing method. However, the *M* – *H* loop of the KBi_2_ sample prepared using a liquid NH_3_ technique (Fig. 2(c)) is similar to that of a α-PdBi_2_ compound (type-II superconductor).^36^ As described later, the values of the lower critical field, *H*_c1_, and the upper critical field, *H*_c2_, were too low, similar to those of KBi_2_.^32^

Fig. 2(d) shows 4π*M* – *H* plots of a KBi_2_ sample prepared using a liquid NH_3_ technique, and recorded at 2.0–3.6 K. As shown in the inset of Fig. 2(d), the lower critical field, *H*_c1_, was evaluated to be 24 Oe at 2.0 K, whereas the *H*_c2_ value was determined to be >500 Oe at 2.0 K; the value of *H*_c1_ was determined from the deviation from the linear line with a slope of −1.0 (as shown in the inset of Fig. 2(d)). A previous report concluded that the KBi_2_ prepared using an annealing method was presumably a type-I superconductor.^32^ Actually, the *M* – *H* loop with a small hysteresis for the KBi_2_ sample^32^ was similar to other type-I superconductors such as YbSb_2_ and LaRhSi_3_.^37–39^ However, the *M* – *H* loop of KBi_2_ prepared in this study (Fig. 2(c)) seems to be different from those of YbSb_2_ and LaRhSi_3_.

Herein, we should discuss why a type-II like superconducting behavior can be seen in the KBi_2_ sample prepared using a liquid NH_3_ technique. The first scenario is based on a type of impurity effect, and the second is based on KBi_2_ prepared using liquid NH_3_, creating an intrinsically bulk type-II superconductor. In a type-II superconductor, 
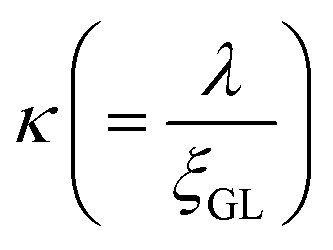
 is larger than 1.0 
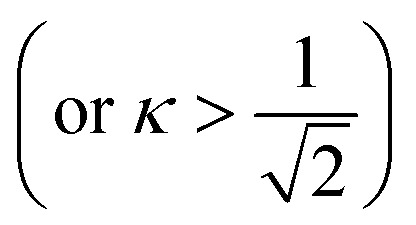
, where 
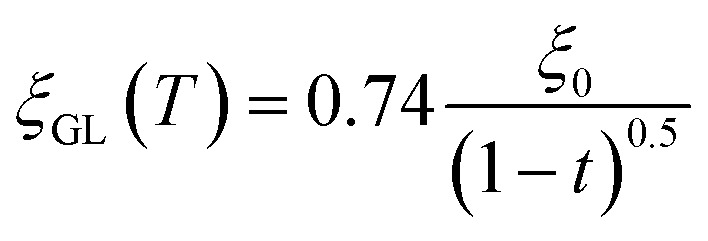
 for a clean limit, and 
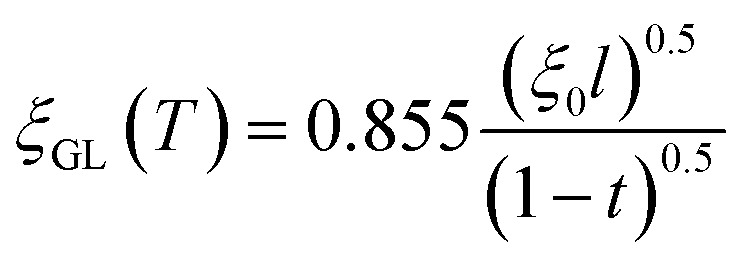
 for a dirty limit, at near *T*_c_; in addition, *t* = *T*/*T*_c_. Here, *ξ*_GL_, *λ*, *ξ*_0_, and *l* refer to the Ginzburg–Landau coherence length, magnetic penetration depth, Pippard coherence length, and mean-free path of a conduction electron, respectively.^40^ Moreover, the magnetic penetration depth under a dirty limit, *λ*_eff_ (or *λ*), is expressed as 
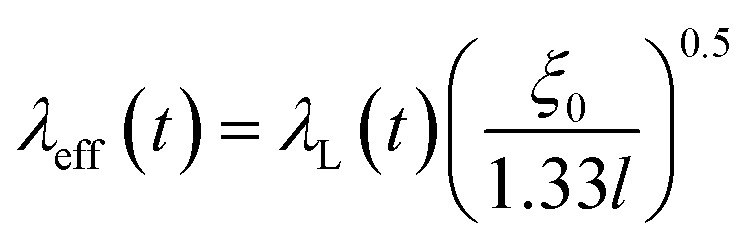
; in addition, *λ*_L_(*t*) is London's penetration depth. Therefore, under a dirty limit, a smaller *l* must lead to a smaller *ξ*_GL_ and greater *λ*, yielding a greater *κ*, *i.e.*, a type-II superconductor. Actually, *l* is governed by the impurity, as indicated by the fact that the conversion of a type-I superconductor into a type-II superconductor is found in the In doping of Pb.^41^ To summarize, the polycrystalline powder sample of KBi_2_ obtained using a liquid NH_3_ technique may result in a small *l* because of the impurity effect. In addition, more detailed experiments such as specific heat measurements at *H* = 0 and *H* ≠ 0 will be significant for determining either a type-I or type-II superconductor, as was achieved for a single crystal of KBi_2_.^32^ In addition, the experiment results will simultaneously indicate whether the KBi_2_ prepared using liquid NH_3_ is a true bulk type-II superconductor, or the result of an impurity effect.

Fig. 3(a) shows the value of upper critical filed, *H*_c2_, *versus T* plot in which *H*_c2_ value at each temperature was defined as *H* value providing 5% of the absolute value of minimum *M*, as indicated in inset of Fig. 3(a). In addition, the experimental *M* – *H* plots for the KBi_2_ sample prepared in this study using high-temperature annealing substantially followed a linear line,^32^ and the value of *H*_c_ (∼160 Oe)^32^ for the KBi_2_ sample at 2.0 K is close to the critical field, 
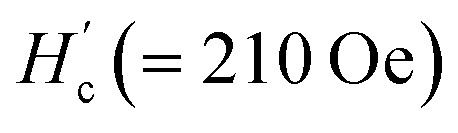
, for KBi_2_ at 2.0 K (see the inset of Fig. 3(a)).

Fig. 3(b) shows the normalized temperature (*t* = *T*/*T*_c_) dependence of the reduced critical field, h*, where h*(*T*) at any temperature (*T*) is given by the equation, 
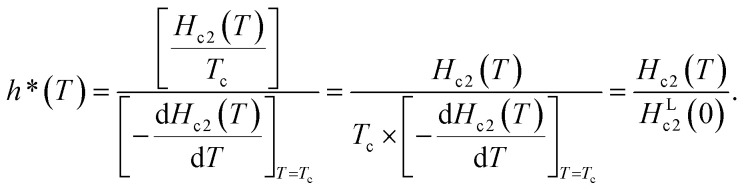
 The value of *H*_c2_(*T*) corresponds to that shown in Fig. 3(a), and the value of 1040 Oe was utilized for *H*^L^_c2_(0); here, *H*^L^_c2_(0) corresponds to 
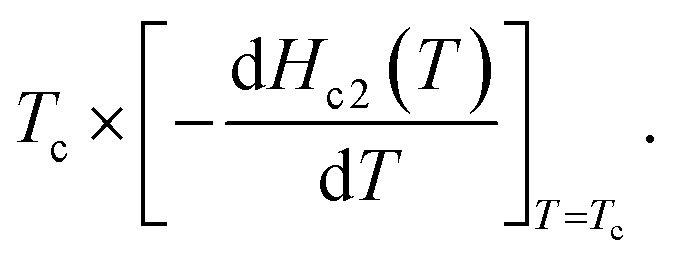
 It should be noted that the dirty limit superconductivity of an *s*-wave corresponds to the Werthamer–Helfand–Hohenberg (WHH) model (h*(0) = 0.69).^42,43^ In addition, the h*(0) value should reach 0.80–0.85 in the case of *p*-wave pairing.^44–49^ As shown in Fig. 3(b), it is difficult to uniquely conclude which model follows the h* – *t* plot between the *s*-wave and *p*-wave. The *H*_c2_ data at a temperature of lower than 2.0 K are required to achieve a unique conclusion. However, the *p*-wave Cooper pair coupling of KBi_2_ seems to lack a positive support. Thus, the superconductivity of KBi_2_ may not comprise a topologically non-trivial nature because the Cooper pair symmetry will be a *p*-wave (with an odd parity) in the case of a topologically non-trivial superconductor.^46–49^ A more detailed study, including a theoretical approach, may be indispensable for pursuing its topological nature. In this study, we further discuss the *H*_c2_ value of the KBi_2_ sample within the framework of an *p*-wave dirty limit model.^42,43^ The *H*_c2_ value was determined to be 720 Oe based on the WHH model, 
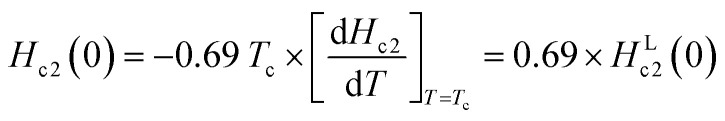
, using an *H*^L^_c2_(0) value of 1040 Oe (Fig. 3(a)).

Moreover, although we tried to determine the value of *H*_c2_ at each temperature, *H*_c2_(*T*), from *T*^onset^_c_ at each *H* (see Fig. 2(b)), and to depict an h* – *t* plot, it was difficult to evaluate *T*^onset^_c_ at high *H*. Therefore, an h* – *t* plot was not created. Based on the WHH model^42,43^
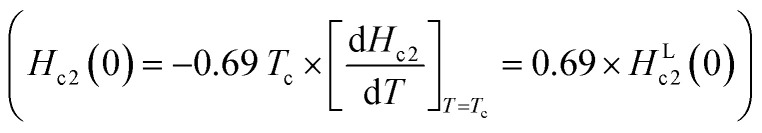
, the *H*_c2_(0) value was determined to be 620 Oe from a linear fitting at near *T*_c_ for the *H*_c2_ – *T* plot (*H*^L^_c2_(0) = 900 Oe in Fig. 3(c)). An *H*_c2_(0) value of 620 Oe may be reasonable owing to an agreement with 720 Oe, as evaluated from the *H*_c2_ – *T* plot shown in Fig. 3(a). As shown in Fig. 3(d), the lower critical field (*H*_c1_(0)) was 37 Oe, as determined from the fitting of the conventional formula, 
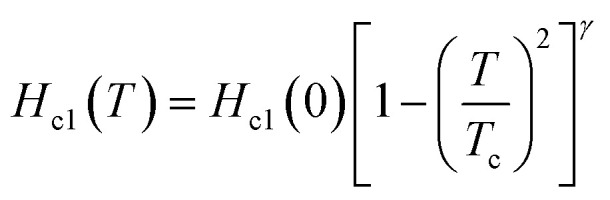
, in which *γ* is ∼1.0. This empirical formula was employed for an evaluation of *H*_c1_ of various superconducting materials including BaBi_3_, CeRu_2_, CaIr_2_, and SrIr_2_.^30,50–52^

In addition, we evaluated *ξ*_GL_ of KBi_2_ to have a value of 73 nm using the expression,^41^
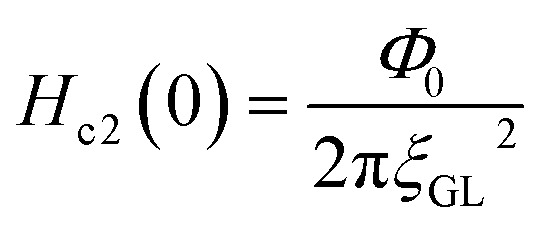
 and the real *H*_c2_(0) to be 620 Oe, where *Φ*_0_ is 2.0678 × 10^−7^ G cm^2^. By contrast, the *λ* value was evaluated as 220 nm using the formula,
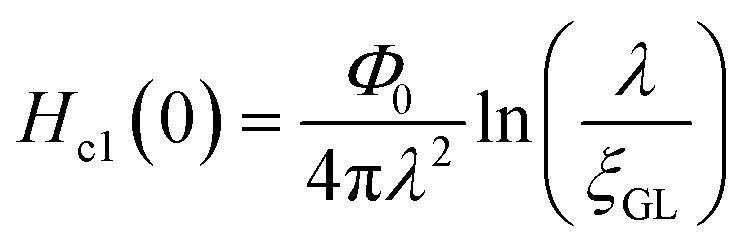
,^40^ and a *H*_c1_(0) value of 37.0 Oe. The value of 
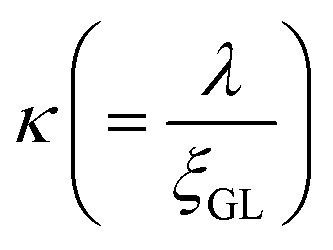
 was ∼3, which is much larger than 
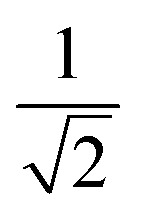
, indicating a type-II superconductor, as described above.

**Fig. 4:**
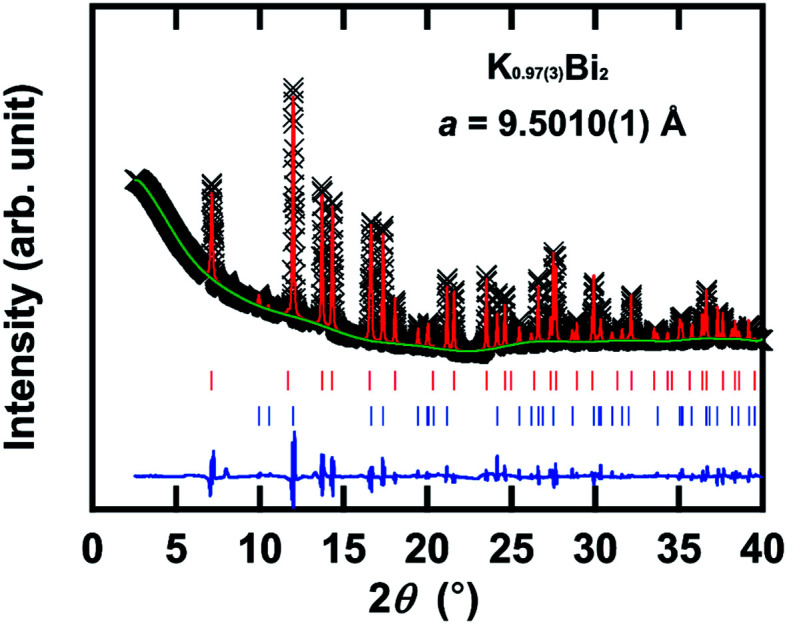
XRD patterns of the KBi_2_ sample prepared using a liquid NH_3_ technique, together with the XRD pattern calculated through the Rietveld refinement. The experimental XRD pattern (black crosses) is shown in each graph, together with the pattern (red solid line) calculated. The red and blue sticks refer to the positions of the predicted Bragg reflections for KBi_2_ (major phase) and Bi (minor phase), respectively. In this graph, the solid blue line corresponds to the difference between the experimental and calculated XRD patterns.

Fig. 4 shows an XRD pattern of the KBi_2_ sample prepared using a liquid NH_3_ technique, along with the XRD pattern reproduced through a Rietveld refinement with a space group of *Fd*3̄*m* (face-centered cubic, no. 227). The lattice constant, *a*, was determined to be 9.5010(1) Å, which is consistent with that (9.5017(1) Å) from a Le Bail analysis (not shown) and *a* = 9.5233(2) Å of KBi_2_ prepared using a high-temperature annealing method.^32^ This phase can be assigned to KBi_2_. Table 1 lists the atomic coordinates of KBi_2_ as determined through a Rietveld refinement. The actual stoichiometry of KBi_2_ determined by a Rietveld refinement was K_0.97(3)_Bi_2_, which was evaluated from the occupancy of K and Bi atoms (see Table 1). The actual stoichiometry is the same as the nominal stoichiometry. In a Rietveld refinement, an additional minor phase that can be assigned to Bi (*R*3̄*m* (rhombohedral, no. 166) and *a* = 4.53440(8) Å and *c* = 11.8310(5) Å in a hexagonal structure) was included. The lattice constants obtained are the same as those of the Bi metal.^53^ In the Rietveld refinement, the experimental and calculated XRD patterns (Fig. 4) showed a good fit. The values of *R*_p_ and w*R*_p_ were 1.55% and 2.87%, respectively.

**Table 1:** Crystal structure of KBi_2_ determined through Rietveld refinement using the space group, *Fd*3̄*m* (no. 227, choice 2). Lattice constant *a* = 9.5010(1) Å

	Occupancy	*x*	*y*	*z*	*B* (Å^2^)
16*c* Bi	1.0	0	0	0	0.72(5)
8*b* K	0.97(3)	0.375	0.375	0.375	1.5(6)

Herein, we discuss whether NH_3_ is included in the KBi_2_ sample prepared using a liquid NH_3_ technique. The *a* value of KBi_2_ prepared in this study is consistent with that of the KBi_2_ sample (*a* = 9.5233(2) Å) prepared using a high-temperature annealing method.^32^ For instance, the *c* value of ammoniated K-doped FeSe ((NH_3_)_*y*_K_*x*_FeSe) was 14.84–16.2 Å,^54,55^ which is larger than that of K_*x*_FeSe, *i.e.*, 14.0367(7) Å, prepared using a high-temperature annealing method.^56^ As a consequence, the KBi_2_ sample prepared using the liquid NH_3_ technique is exactly KBi_2_ without NH_3_. It can be concluded that the liquid NH_3_ technique can exactly yield a KBi_2_ sample in the same manner as a high-temperature annealing method.

**Fig. 5:**
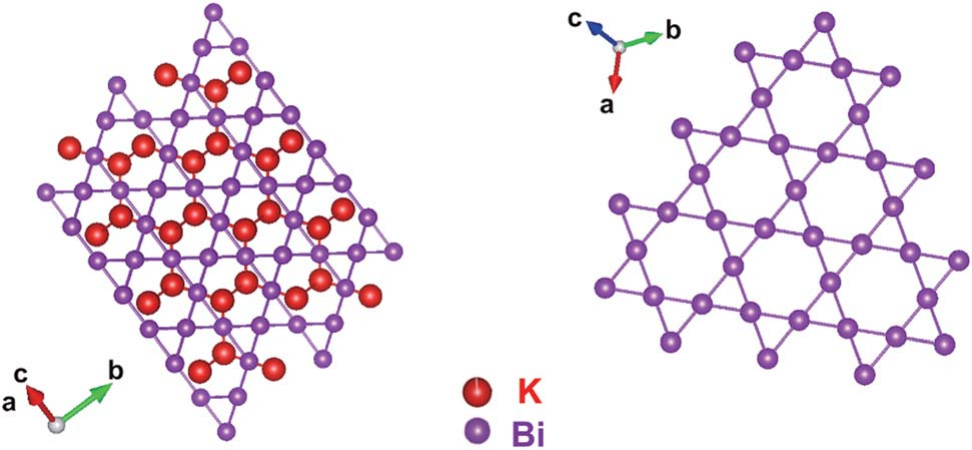
Schematic representation of crystal structure of KBi_2_, which was determined using a Rietveld refinement (Fig. 4(b)).

A schematic representation of the crystal structure of KBi_2_ depicted with the atomic coordinates determined from the Rietveld refinement for an XRD pattern (Fig. 4) is shown in Fig. 5. As shown in the projection from the [111] direction, the Bi atoms form a Kagomé net, which is the same as a previously reported crystal structure.^32^ The atoms of K are linked to the Bi atoms in a 3D network, as shown in Fig. 5. The crystal structure obtained is the same as that reported previously.^32^ As a consequence, throughout this study, it was demonstrated that the liquid NH_3_ technique can easily produce a crystalline powder sample of KBi_2_ in the same manner as used in a high-temperature annealing method. The value of *T*_c_ and the crystal structure are consistent with those previously reported.^32^

## 4. Conclusions

A new protocol for the preparation of superconducting KBi_2_ was completely verified through this study, *i.e.*, a liquid NH_3_ technique is available for the preparation of a crystalline powder of superconducting KBi_2_. The *T*_c_ value of KBi_2_ prepared in this study was consistent with that reported previously.^32^ The crystal structure was also the same as that described elsewhere.^32^ Regardless of the preparation when using liquid NH_3_, the inclusion of NH_3_ or amide was not confirmed in the KBi_2_ sample. Actually, the magnetic properties of a polycrystalline KBi_2_ sample prepared using a liquid NH_3_ technique were different from those of the single-crystal KBi_2_ prepared through a high-temperature annealing method.^32^ The reason why the former is a type-II superconductor and the latter is type-I remains questionable, although pursuing it may be extremely significant from the chemistry viewpoint of material synthesis as well as the physics perspective of a superconductor.

Because the liquid NH_3_ technique is extremely effective and simple in terms of the preparation of KBi_2_, various metal–Bi compounds may easily be fabricated using the technique, leading to an elucidation of their superconducting behavior and quantum/topological properties. Admittedly, this type of material is interesting owing to the suggested topological nature such as a Dirac semimetal.^29,30^ At the present stage, the topological nature of the superconducting KBi_2_ sample was definitely not demonstrated. The pursuit of a topological nature in metal–Bi compounds must be continued; our approach regarding the preparation of a Bi-rich compound using a liquid NH_3_ technique is currently being used to produce various alkali–Bi compounds such as LiBi, NaBi, RbBi_2_, and CsBi_2_ as superconductors.^57^

To summarize, the development of an effective, simple, and exact preparation of metal–Bi compounds as described in this paper is significant for a detailed study on the superconducting behaviors of their compounds. The next target is to synthesize new superconducting metal–Bi compounds that have yet to be prepared using a high-temperature annealing method.

## Author contributions

Huan Li, Yanan Wang, and Yutaro Aoki prepared the KBi_2_ sample using a liquid NH_3_ technique. In addition, Huan Li, Yanan Wang, and Yutaro Aoki carefully measured the magnetic behavior of the KBi_2_ sample, under cooperation with Takashi Kambe. Huan Li, Yanan Wang, Xiaofan Yang, Tomoya Taguchi, Akari Miura, Ai Suzuki, Lei Zhi, and R. Eguchi measured the X-ray diffraction patterns (XRDs) of the KBi_2_ sample and analyzed the data using Le Bail and Rietveld analysis methods. The XRD measurement system using synchrotron radiation was designed by Yen-Fa Liao and Hirofumi Ishii, which allowed the suitable results of this study to be achieved. Yoshihiro Kubozono supervised all of the measurements and data analyses conducted in this study and prepared the manuscript under discussions with Hidenori Goto and Huan Li. Saki Nishiyama suggested the idea of this study, and the preliminary experiment was conducted by Yoshihiro Kubozono. All coauthors finally reviewed the manuscript.

## Conflicts of interest

The authors declare no competing financial interests.

## Supplementary Material
